# A case of Page kidney due to an encapsulated perirenal hematoma improved by capsulectomy

**DOI:** 10.1002/iju5.12837

**Published:** 2025-01-30

**Authors:** Tomoki Takeda, Atsushi Okada, Keitaro Iida, Toshiki Etani, Kazumi Taguchi, Taku Naiki, Akihiro Nakane, Yasue Kubota, Yukihiro Umemoto, Noriyasu Kawai, Yutaro Hayashi, Takahiro Yasui

**Affiliations:** ^1^ Department of Nephro‐urology Nagoya City University Graduate School of Medical Sciences Nagoya Aichi Japan; ^2^ Department of Pediatric Urology Nagoya City University Graduate School of Medical Sciences Nagoya Aichi Japan

**Keywords:** capsulectomy, encapsulated perirenal hematoma, hypertension, Page kidney, renal hypoperfusion

## Abstract

**Introduction:**

Page kidney causes secondary hypertension due to external compression of the renal parenchyma and renal hypoperfusion.

**Case presentation:**

A 21‐year‐old healthy man was diagnosed with hypertension during a medical examination 3 months after he had gotten a bruise at the right lumbar region. The patient's blood pressure was 176/120 mmHg. Computed tomography showed a large encapsulated cystic lesion around the right kidney and severe external compression of the renal parenchyma. Dimercaptosuccinic acid renal scintigraphy revealed decreased right split renal function. Therefore, the patient was diagnosed with a Page kidney. Percutaneous drainage of the hematoma was performed. However, the hematoma enlarged again. Capsulectomy was subsequently performed. The pathological findings revealed capsule fibrosis and hyperplasia of the capillaries in the inner capsule layer. After capsulectomy, the patient's blood pressure normalized.

**Conclusion:**

Blood leakage from the fibrotic capsule likely maintained the hematoma. Capsulectomy is recommended in cases involving an encapsulated perineal hematoma.

**Abbreviations & Acronyms**
CSH: chronic subdural hematomas
CT: computed tomography
DMSA: dimercaptosuccinic acid
PRA: plasma renin activity
RAAS: renin–angiotensin–aldosterone system

**Keynote message**

We report a case of Page kidney due to an encapsulated perirenal hematoma. After capsulectomy, the patient's blood pressure normalized. The discussions on the mechanism of hematoma formation and the appropriate treatment for Page kidney were based on the pathological findings of the hematoma capsule.

## Introduction

Page kidney is defined as the external compression of the renal parenchyma, typically caused by a perirenal hematoma. This results in renal ischemia and secondary hypertension.^1^, ^2^ We report a case of Page kidney due to an encapsulated perirenal hematoma.

## Case report

A 21‐year‐old man was diagnosed with hypertension during a medical examination 3 months after he had gotten a bruise at the right lumbar region while snowboarding. Ultrasound and CT showed a cystic lesion around the right kidney, and he was referred to our hospital in March 2016. The findings of the urinalysis and blood test were unremarkable.

**Fig. 1:**
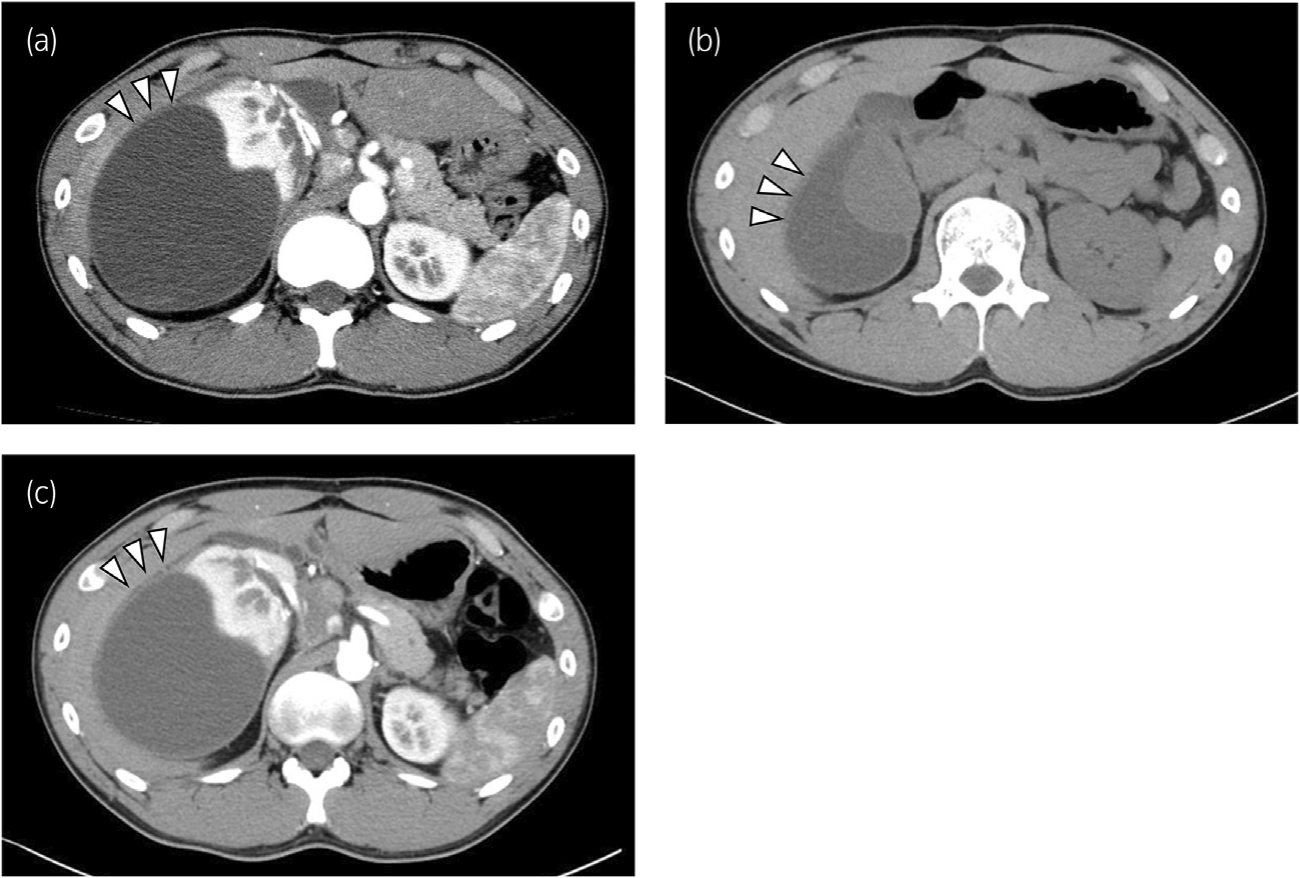
Fig. 1a,c are contrast CT images. Fig. 1b is a plain CT image. (a) CT showed a large encapsulated cystic lesion (measuring approximately 14 × 10 × 12 cm) around the right kidney (white arrowheads), with severe external compression of the renal parenchyma. (b) One month after of percutaneous drainage of the right perirenal hematoma, CT showed volume reduction of the hematoma (white arrowheads) and improvement of external compression of the renal parenchyma. (c) Six months after percutaneous drainage, the right perirenal hematoma enlarged again (white arrowheads).

CT showed a large encapsulated cystic lesion around the right kidney (measuring approximately 14 × 10 × 12 cm) and severe external compression of the renal parenchyma (Fig. 1a). DMSA renal scintigraphy revealed a right split renal function of 15.6%. The PRA was 2.9 ng/mL/h. Before treatment, the PRA was examined thrice, and the results were all normal. In addition, the other hormone levels were normal (aldosterone 121 pg/mL, adrenocorticotropic hormone 18.7 pg/mL, cortisol 7.3 μg/dL, and urinary vanillylmandelic acid 2.6 mg/gCr). CT angiography did not detect renal artery stenosis. Therefore, the patient was diagnosed with a Page kidney due to a right perirenal hematoma.

**Fig. 2:**
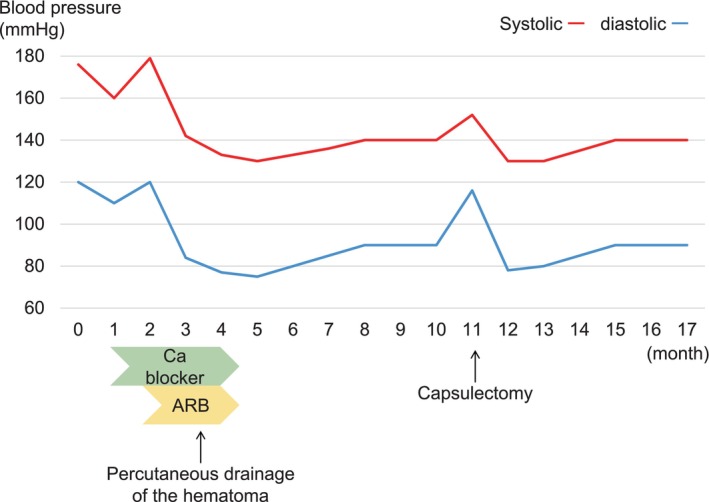
Clinical course. The horizontal axis of the figure shows the month since the first visit to our hospital. ARB, angiotensin receptor blocker, Ca blocker, calcium blocker.

His hypertension improved with medication (Fig. 2). Percutaneous drainage of the hematoma with ultrasonic guidance was performed in December 2016, and 780 mL of yellowish‐brown liquid was aspirated. Percutaneous drainage was performed via a single puncture. Although we attempted to place a drainage tube into the cyst, the hard cyst capsule prevented its insertion. One month after percutaneous drainage, CT showed volume reduction of the hematoma (Fig. 1b). The patient's blood pressure normalized without medication. However, the hematoma enlarged again (Fig. 1c), and his blood pressure gradually increased (Fig. 2). Therefore, capsulectomy was performed in August 2017.

**Fig. 3:**
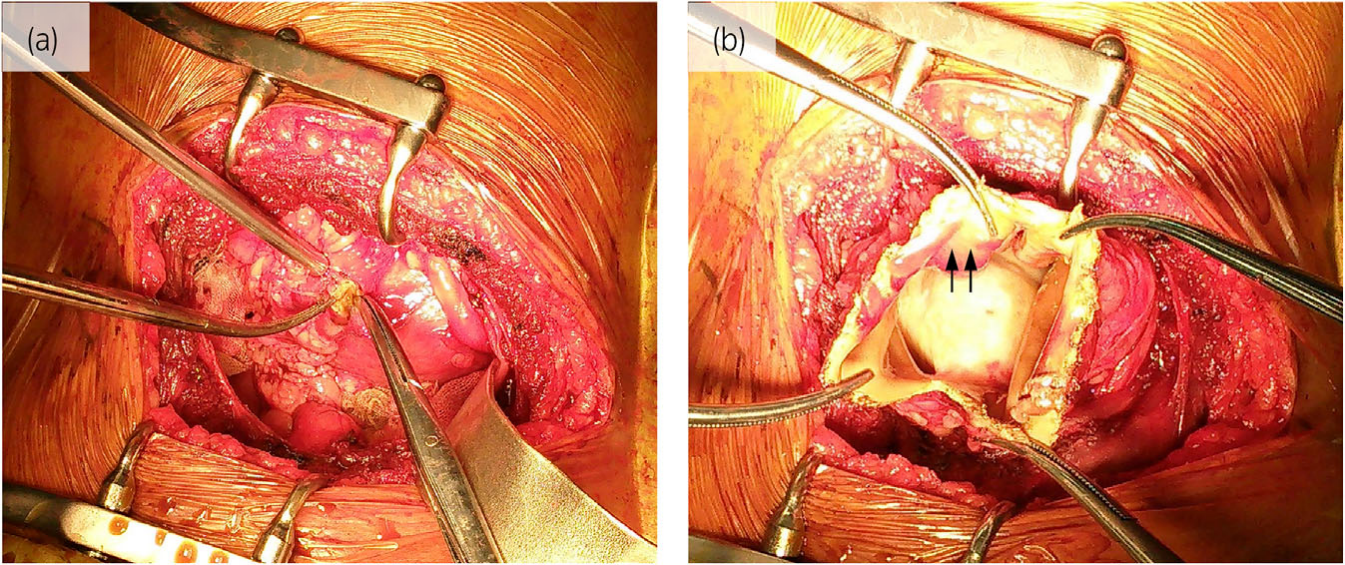
(a) Before fenestration of the right perirenal hematoma. (b) After fenestration. A smooth inner surface of the capsule was observed (arrows).

**Fig. 4:**
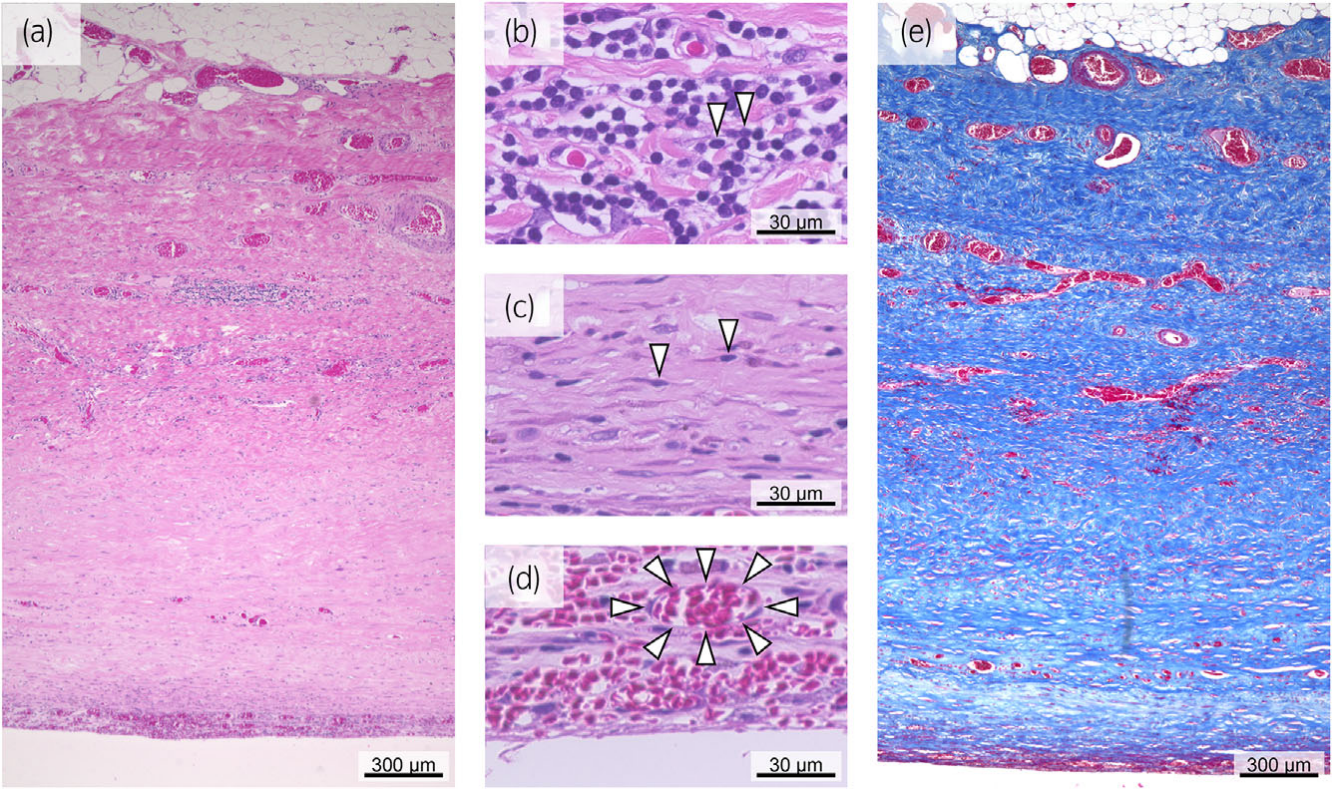
(a) Hematoxylin and eosin staining showed the entire thickness of the capsule. (b) Inflammatory cells (white arrowheads). (c) Fibroblasts (white arrowheads) and collagen fibers. (d) Hyperplasia of capillaries (white arrowheads) in the inner layer of the capsule. (e) Masson trichrome staining. The blue color showed that most of the capsule was occupied by collagen fibers.

We performed open surgery using the translumber approach because adhesions and a small working space were anticipated. After the hematoma had been fenestrated, the smooth inner surface of the capsule was observed (Fig. 3). The absence of bleeding from the inner surface was confirmed, and the capsule was removed. Pathological findings showed capsule fibrosis (Fig. 4a). Inflammatory cells and fibroblasts were found in the middle capsule layer (Fig. 4b,c), and capillary proliferation was noted in the inner layer (Fig. 4d). Masson trichrome staining revealed that most of the capsules were occupied by collagen fibers (Fig. 4e).

After capsulectomy, his blood pressure normalized without medication (Fig. 2). Three months after the surgery, CT showed volume reduction of the hematoma, and DMSA renal scintigraphy revealed improvement of the right split renal function (37.4%). Serum creatinine levels were normal before the capsulectomy, and no change in serum creatinine levels was observed postoperatively.

## Discussion

In 1939, Page IH demonstrated the occurrence of hypertension by wrapping kidneys in cellophane and compression of the renal parenchyma in an animal model.^2^ In 1995, Engel WJ and Page IH reported a case of hypertension due to external compression of the renal parenchyma caused by a perirenal hematoma.^3^ Since then, hypertension due to external compression of the renal parenchyma has been called Page kidney. The causes of Page kidney include perirenal hematomas following blunt trauma,^4^ extracorporeal shock wave lithotripsy,^5^ renal biopsy,^6^ and renal transplantation.^7^

The perirenal hematomas involved in Page kidney are large and capsulated.^8^ However, the mechanism of hematoma formation is unknown. CSH are also capsulated. In CSH, plasma‐fibrin forms the capsule, and the breakdown products, derived from solid blood elements, induce chronic inflammation. This promotes capillary proliferation, and capillary blood leakage results in large hematoma formation.^9^ In this case, the perirenal hematoma had a fibrotic capsule with inflammatory cells and capillaries. The mechanism of capsule formation in the Page kidney was reportedly similar to that of CSH.

In Page kidney, the activation of the RAAS due to renal hypoperfusion causes hypertension.^1^ This supports the involvement of RAAS in this case. However, significant hyperreninemia was not observed. Renovascular hypertension is also caused by renal hypoperfusion. In renovascular hypertension, the onset of hypertension due to the sympathetic activation has been previously reported.^10^ In Page kidney, mechanisms aside from RAAS activation may cause hypertension.

The treatment options for Page kidney include percutaneous drainage,^11^ hematoma fenestration,^12^ and nephrectomy.^13^ In this case, percutaneous drainage was performed. However, the effect of hematoma reduction was temporary. Blood leakage from the fibrotic capsule likely increased the volume of the hematoma. In addition, the inner surface of the fibrotic capsule did not adhere to each other by percutaneous drainage because the inner surface, observed during capsulectomy, was smooth.

In a report by Zuh, no recurrences were observed in 10 patients with perineal hematomas, treated via capsulectomy.^14^ In this case, capsulectomy prolonged the hematoma reduction effect and improved the patient's blood pressure. In cases where hypertension fails to improve with capsulectomy, nephrectomy should be considered. However, less invasive treatments, such as resumption of medication, should be considered before nephrectomy. Additionally, in this case, split renal function improved, and the kidney was preserved as much as possible.

## Conclusions

We reported a case of Page kidney due to an encapsulated perirenal hematoma, alleviated by capsulectomy. The capsule of the perirenal hematoma was fibrotic with capillary hyperplasia in the inner layer. Blood leakage from the fibrotic capsule likely maintained the hematoma. If the perirenal hematoma is encapsulated and percutaneous drainage was ineffective, capsulectomy is recommended.

## Author contributions

Tomoki Takeda: Writing – original draft; conceptualization. Atsushi Okada: Conceptualization; supervision. Keitaro Iida: Review and editing. Toshiki Etani: Review and editing. Kazumi Taguchi: Review and editing. Taku Naiki: Review and editing. Akihiro Nakane: Review and editing. Yasue Kubota: Supervision. Yukihiro Umemoto: Supervision. Noriyasu Kawai: Supervision. Yutaro Hayashi: Supervision. Takahiro Yasui: Supervision.

## Conflict of interest

Takahiro Yasui is an Editorial Board member of the International Journal of Urology and a coauthor of this article. To minimize bias, he was excluded from all editorial decision‐making related to the acceptance of this article for publication.

## Approval of the research protocol by an Institutional Reviewer Board

This case report was approved by the Ethics Committee of Nagoya City University Graduate School of Medical Sciences, Nagoya, Japan (60‐22‐0092).

## Informed consent

Not applicable.

## Registry and the Registration No. of the study/trial

Not applicable.
